# The developing visual system: A building block on the path to autism

**DOI:** 10.1016/j.dcn.2025.101547

**Published:** 2025-03-12

**Authors:** Jessica B. Girault

**Affiliations:** a Carolina Institute for Developmental Disabilities, University of North Carolina at Chapel Hill, Chapel Hill, NC 27599, USA; b Department of Psychiatry, University of North Carolina at Chapel Hill, Chapel Hill, NC 27599, USA

**Keywords:** Infant, Brain, Neuroimaging, Autism, Development

## Abstract

Longitudinal neuroimaging studies conducted over the past decade provide evidence of atypical visual system development in the first years of life in autism spectrum disorder (ASD). Findings from genomic analyses, family studies, and postmortem investigations suggest that changes in the visual system in ASD are linked to genetic factors, making the visual system an important neural phenotype along the path from genes to behavior that deserves further study. This article reviews what is known about the developing visual system in ASD in the first years of life; it also explores the potential canalizing role that atypical visual system maturation may have in the emergence of ASD by placing findings in the context of developmental cascades involving brain development, attention, and social and cognitive development. Critical gaps in our understanding of human visual system development are discussed, and future research directions are proposed to improve our understanding of ASD as a complex neurodevelopmental disorder with origins in early brain development.

## Highlights

• Infant neuroimaging suggests atypical visual system development in ASD.
• A developmental cascade framework is proposed for visual system development in ASD.
• Future directions for study in ASD and typical development are proposed.

## 1 Introduction

Autism spectrum disorder (ASD) is a lifelong condition diagnosed in 2–3 % of the U.S. population (Maenner et al., 2023) that requires varying levels of support, with estimated costs of care expected to exceed $461 billion annually in the by 2025 (Leigh and Du, 2015). While the median age of diagnosis is approximately four years (Maenner et al., 2023), early signs of ASD emerge over the later part of the first and second years of life (Shen and Piven, 2017), spurring calls for early, presymptomatic (Grzadzinski et al., 2021, Wolff and Piven, 2020) interventions in the first years of life. While there has been progress in demonstrating feasibility and acceptability of presymptomatic (before age 18 months) behavioral interventions (Rogers et al., 2014, Team et al., 2013), these studies have yet to demonstrate robust results and there is much to learn about when and how to intervene during heightened periods of plasticity that may confer the greatest long-term benefit for adaptive outcomes.

One of the greatest risk factors for ASD is having a sibling with a diagnosis (Lord et al., 2020), reflecting autism’s high heritability (Sandin et al., 2017) driven by a complex genetic architecture involving both rare and common inherited variation (Cirnigliaro et al., 2023). Recurrence rates in families are approximately 20 %, with 1 in 5 younger siblings of ASD children meeting criteria for ASD themselves by three years of age (Ozonoff et al., 2024, Ozonoff et al., 2011). Infant siblings represent a subset of the population at high likelihood (HL) for developing ASD and have been studied widely over the past two decades using prospective longitudinal designs. Studies have mapped developmental trajectories in brain and behavior to reveal that differences in brain development are present in the first year of life and precede or coincide with early differences in sensory, motor, and language behaviors – all of which are detectable well before consolidation of symptoms into the full syndrome of ASD not typically observed until the later part of the first and second years of life (Girault and Piven, 2020).

Several new reports from infant and toddler magnetic resonance imaging (MRI) studies have converged on the atypical development of the visual system in ASD, mirroring a growing literature on visual system differences in older children and adults with ASD (Berto et al., 2022, Buch et al., 2023, Hong et al., 2019, Ilioska et al., 2023, Schelinski et al., 2024, Spiegel et al., 2019). This aligns with postmortem and transcriptomic analyses indicating robust differential gene expression in the visual cortex in ASD (Gandal et al., 2022), and recent work linking familial markers of inherited genetic liability for ASD to individual variation in the structure and function of components of the visual system (Girault et al., 2022). These findings may explain the well documented differences in visual attention behaviors present in the infancy and toddler periods in ASD (Bradshaw et al., 2019, Elison et al., 2013a, Elsabbagh et al., 2013, Jones and Klin, 2013), making the visual system an important neural phenotype along the path from genes to behavior that deserves further study.

In light of this prior work, this article outlines evidence for a potential developmental cascade involving early differences in visual circuit development that may subserve differences in visually guided behaviors, alter experience-dependent brain development and ultimately give rise to downstream differences in brain and behavior observed in ASD (Girault et al., 2022, Piven et al., 2017). The current state of the literature regarding visual system development in the first years of life in ASD is presented, with a focus on studies using MRI which are most abundant in the literature to date and have the required spatial resolution to assess underlying circuitry. Neurobiological findings are discussed in the context of emerging differences in visual attention and social behavior across this period and integrated in a developmental cascades conceptual framework linking visual brain development to differences in infant behavior and ultimately the later emergence of the autistic phenotype. Gaps in our understanding of both typical brain development and brain development in ASD are highlighted, and key directions for future studies are proposed. While the focus here is on the visual system given the growing evidence of converging findings across neuroimaging modalities, cohorts, and age ranges, as well as evidence of links to genetic factors, it should be noted that differences in other circuits/systems (e.g., other sensory circuitry, cerebrospinal fluid neurobiology) have been reported during infancy in ASD (Riva et al., 2018, Shen et al., 2018, Shen et al., 2017, Shen et al., 2013) and likely also play an important role in the development of ASD that warrants further study. As such, the visual system is presented here as one possible building block on the developmental pathway to ASD; other potential pathways are discussed elsewhere (Bradshaw et al., 2022a).

## 2 Visual system development during infancy in ASD

Vision is a dominant perceptual ability in humans and the visual cortex is the most neuronally dense (Collins et al., 2016, Collins et al., 2010, Gandal et al., 2022) and one of the most highly interconnected areas in the brain. Visual input is transmitted from the retina to the thalamus (lateral geniculate nuclei) to the primary visual cortex, where it is then routed along the dorsal and ventral streams for further processing (Corbetta et al., 2008). Many other brain regions share direct connections to the visual cortex and are responsible for processing visual information and modulating behavioral responses. This includes, for example, the amygdala which is in part responsible for regulating the allocation of visual attention (Adolphs and Spezio, 2006), as well as other subcortical/midbrain circuitry, including the superior colliculus and pulvinar which may play a particularly important role in visual processing during infancy (Johnson, 2005, Johnson et al., 2015a, Johnson et al., 2015b). In this review, these “extended” circuit components (i.e., amygdala) are referred to as part of the visual system though they may fall outside of the canonical visual circuitry often described in the adult literature. This is done to offer a wholistic interpretation of the findings (i.e., implications for behaviors like visual attention/orienting) and in appreciation of the fact that much is unknown about the neural circuitry that supports visual processing and attention across early infancy in humans.

The first year of life marks the most rapid period of postnatal visual system development in the human lifespan, defined by extensive circuit maturation and refinement at both the cellular (Siu et al., 2017, Siu and Murphy, 2018) and network level (Gao et al., 2016, Gilmore et al., 2018), and coinciding with substantial changes in visual perception and attention (Braddick and Atkinson, 2011, Hendry et al., 2019). These maturational processes occur in both a genetically-driven and experience-dependent fashion (Jandó et al., 2012, Li et al., 2022) and include rapid growth of the occipital cortex (Li et al., 2022, Lyall et al., 2015), as well as microstructural changes in white matter in visual cortical areas and underlying fiber pathways, driven largely by myelination (Chen et al., 2022, Deoni et al., 2015, Dubois et al., 2008, Natu et al., 2021). Studies of resting state functional connectivity show refinements in broad-scale visual networks across the first years of life (Gao et al., 2016), while recent advances in awake task-based MRI methods for infants have demonstrated that cortical visual areas may be more functionally mature than previously recognized. Areas that process motion are operative by 5–7 weeks of age (Biagi et al., 2023, Biagi et al., 2015), the retinotopic organization of the primary visual cortex is in place by 5 months (Ellis et al., 2021), and the general organization of face processing areas is reminiscent of that in adults by 4–6 months (Deen et al., 2017). However, these studies have been reported in relatively few infants and require replication.

**Fig. 1:**
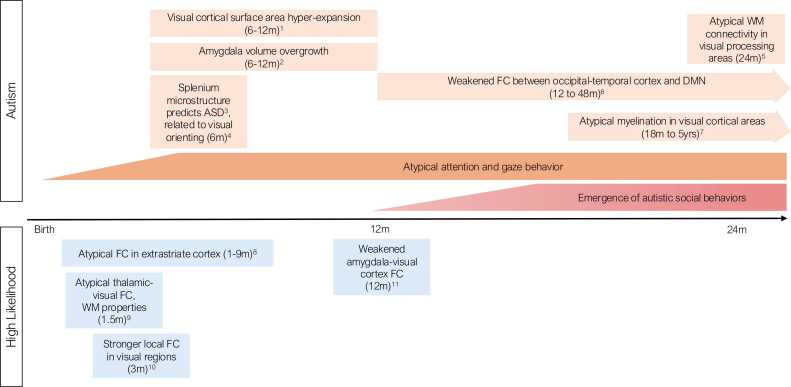
Developmental timeline of visual system differences in ASD and HL infants from birth to 24 months. Top panel (“Autism”): Differences in visual system circuitry in ASD during infancy and toddlerhood are shown (pastel orange) across the period from birth to 24 months (and beyond, indicated by arrows). General timing of the emergence of atypical visual attention and gaze behaviors (dark orange) and autistic social deficits (red) are shown for comparison. Bottom Panel (“High Likelihood”): Differences in visual system are shown for studies that compare high likelihood to typical development (blue). References for each neuroimaging study are shown as superscripts: 1. Hazlett et al., (2017)*; 2. Shen et al., (2022)*; 3. Wolff et al., (2017)*; 4. Elison et al., (2013a)*; 5. Lewis et al., (2014)*; 6. Lombardo et al., (2019); 7. Chen et al., (2022); 8. Rolison et al., (2021); 9. Nair et al., (2021); 10. Ciarrusta et al., (2020); 11. Liu et al., (2024)*. References with an asterisk indicate studies conducted on the same cohort of infants. FC = functional connectivity MRI; WM = white matter.

It is during this same period of rapid postnatal growth that differences in visual system development are first detectable in infants later diagnosed with ASD (Fig. 1). These brain differences are apparent across imaging modalities, generally appear within the first year of life, and implicate multiple neurobiological mechanisms.

### 2.1 Cortical hyper-expansion

In a landmark structural MRI study of infant siblings, Hazlett and colleagues reported that between 6 and 12 months of age, the surface area of the cortex expands at a faster rate in HL infants later diagnosed with ASD (HL-ASD) compared to both HL infants who did not develop ASD by age 2 (HL-Neg) and typically developing (TD) infants with no family history of ASD (Hazlett et al., 2017). Notably, this faster growth rate, or “hyper-expansion”, of the cortex was most robust in several regions along the dorsal and ventral visual pathways, including the bilateral cuneus (striate cortex, primary visual areas), bilateral middle occipital gyrus, putatively overlapping with extrastriate motion processing area MT/V5 (Biagi et al., 2023, Biagi et al., 2015), right lingual gyrus (striate/extrastriate areas), and left inferior temporal gyrus (occipito-temporal cortex). This cortical hyper-expansion in the first year of life correlated with and preceded the later overgrowth of global brain volume in the second year of life (Hazlett et al., 2017), which is a well-documented neuroimaging phenotype in ASD that persists into adulthood (Piven et al., 1996, Piven et al., 1995, Piven et al., 1992, Yankowitz et al., 2020). In line with this, Ohta and colleagues conducted an MRI study in 3-year-old boys with ASD and reported increased surface area in primary sensory (including visual), association, and prefrontal cortices (Ohta et al., 2016). This suggests that mechanisms governing cortical hyper-expansion in areas important for visual processing in the first year of life precede widespread differences in cortical surface expansion and brain overgrowth in ASD. Though, it will be critical to replicate these findings, as, to date, no other studies of cortical maturation exist across this age range in HL samples for comparison.

### 2.2 Atypical development of white matter

Diffusion tensor imaging studies have documented atypical white matter developmental trajectories during infancy in ASD, with HL-ASD infants exhibiting slowed trajectories of white matter development from 6 to 24 months of age (Wolff et al., 2012), potentially indicative of altered or delayed myelination. Several studies report differences in white matter circuitry supporting visual processing in ASD. White matter fractional anisotropy at 6 months of age in the splenium, a major fiber bundle critical to facilitating interhemispheric communication between visual areas (Putnam et al., 2010), is predictive of later ASD diagnosis (Wolff et al., 2017) and differentially related to visual orienting ability in HL-ASD infants compared to TD infants (Elison et al., 2013a). Atypical network structure has also been reported among areas responsible for visual processing and audio-visual integration in 24-month-old HL-ASD infants (Lewis et al., 2014), with many of the same areas also exhibiting differences in network integrity in the first year of life (Lewis et al., 2017).

A small study of infants at HL for ASD suggests these differences in visuo-occipital tracts may emerge even earlier in development, reporting that atypical thalamic-occipital white matter features (increased mean diffusivity) were apparent in HL infants compared to controls at 6 weeks of age (Nair et al., 2021). Atypical developmental trajectories of white matter, and particularly myelination, among regions responsible for visual processing may also persist later in toddlerhood and preschool. Using T1/T2 ratio maps, Chen et al., reported significant age-related differences in cortical myelination between ASD and TD children (ages 1.5–5 yrs), such that the ASD group did not show the same age-related increases in myelination among visual, posterior cingulate, and precuneus regions as observed in TD, suggesting altered, or slowed, development of myelination within these regions in ASD (Chen et al., 2022). This aligns with diffusion tensor imaging findings, suggesting that overall trajectories of white matter maturation are slowed or delayed in ASD across the late infancy to toddlerhood/preschool period, with differences in visual system white matter emerging early and persisting.

### 2.3 Overgrowth and connectivity of the amygdala

The amygdala has long been implicated in ASD (Baron-Cohen et al., 2000, Chevallier et al., 2012, Pelphrey et al., 2011) and is thought to play a central role in directing visuospatial attention during face processing by modulating visual cortical areas (Adolphs and Spezio, 2006) via direct neuronal projections (Amaral and Price, 1984). Recent evidence suggests that the amygdala develops atypically in HL-ASD infants in the first year of life, with downstream consequences for social impairments. Shen and colleagues reported that the amygdala experiences a period of rapid volumetric overgrowth measured on MRI between 6 and 12 months of age in HL-ASD infants, with growth rates correlating with social behaviors observed in HL-ASD infants at 24 months, such that faster rates of change in amygdala growth were associated with greater impairment (Shen et al., 2022). In a follow-up functional MRI study in an overlapping sample, amygdala functional connectivity in 12-month-old infants was examined, hypothesizing that connectivity differences would be present in HL infants, between the amygdala and visual cortex (which also undergoes a period of rapid growth from 6 to 12 months (Hazlett et al., 2017)). HL infants were found to have weaker connectivity between the right amygdala and left visual cortex, with amygdala-visual cortex connectivity being associated with motor and communication abilities (Liu et al., 2024) that are already atypical by this age in HL infants who later develop ASD. While this functional connectivity study did not examine HL-ASD infants (due to sample size constraints), findings did align with a study reporting weakened amygdala-visual cortex connectivity in older ASD children (Fishman et al., 2018), suggesting that differences in connectivity between the amygdala and visual areas may persist beyond infancy.

### 2.4 Differences in functional connectivity

Most infant MRI studies of functional connectivity published to date include group analyses comparing HL infants with infants born into families with an older TD sibling (no history of ASD), and do not include diagnostic outcome, either because infants had not yet reached the age of diagnosis, or sample sizes were insufficient for analyses split by diagnosis (Liu et al., 2024). These HL studies provide important insights into how genetic liability for ASD impacts the developing brain, with many of the findings centering around atypical development in visual system functional connectivity. As mentioned above, HL infants exhibit weakened amygdala-visual cortex connectivity by the first birthday (Liu et al., 2024). Studies have also reported earlier differences in visual system connectivity among HL infants, including overconnectivity between the thalamus and occipital cortex at 6 weeks of age (Nair et al., 2021), atypical trajectories of functional connectivity between the extrastriate cortex and other brain regions from 1 to 9 months of age (Rolison et al., 2021), and greater local connectivity in visual and sensory regions at 3 months of age (Ciarrusta et al., 2020). These findings align with evidence that weaker connectivity between visual and other networks – including the default mode network (DMN) – at 6 months of age, is related to greater genetic liability for ASD (Girault et al., 2022) as indicated by higher levels of autistic social traits in older ASD siblings (Girault et al., 2020). Weaker connectivity between the visual networks and the DMN has been linked to fewer bids for joint attention in a combined sample of HL and TD infants (Eggebrecht et al., 2017) and has been reported in ASD toddlers with deficits in visual-social engagement (Lombardo et al., 2019). Taken together, these findings suggest that HL infants exhibit atypical development of functional brain networks involved in visual processing, that may have implications for visual attention and downstream social development.

### 2.5 Summary and developmental timeline of visual system differences in ASD

Differences in the visual system in ASD, and HL infants, unfold across the first year of life and are present prior to the emergence of global brain overgrowth and overt ASD symptoms that appear in the second year (Piven et al., 2017), making it perhaps one of the earliest targetable circuits for intervention. As outlined in Fig. 1, atypical development of white matter in visual-occipital tracts is detectable in HL-ASD infants by 6 months of age and persists into toddlerhood, defined by delayed or protracted development likely driven by differences in myelination (Chen et al., 2018), with impacts on the integrity of white matter networks involved in visual processing (Lewis et al., 2017, Lewis et al., 2014). In the latter part of the first year of life (6–12 months of age), the cortex hyper-expands in HL-ASD infants, particularly in regions along the dorsal and ventral visual pathways (Hazlett et al., 2017), concurrent with the overgrowth of the amygdala (Shen et al., 2022). At the end of this period of rapid growth the amygdala and visual cortex exhibit weakened functional connectivity among HL infants, suggesting altered signaling with implications for cognitive and behavioral development (Liu et al., 2024). Weakened connectivity between the visual network and other networks (e.g., DMN) is apparent by toddlerhood in children with ASD who exhibit deficits in visual-social attention (Lombardo et al., 2019). Studies of HL infants in the neonatal period suggest that differences in visual system functional connectivity (Ciarrusta et al., 2020, Nair et al., 2021, Rolison et al., 2021) and white matter properties (Nair et al., 2021) may be present as early as the first months of life, though the links to ASD must be confirmed in future studies incorporating diagnostic group comparisons and symptom outcomes.

These findings in infancy mirror a growing body of evidence of differences in the visual system documented in older children and adults with ASD using MRI, across a range of affectation, including atypical functional connectivity involving visual networks (Berto et al., 2022, Buch et al., 2023, Hong et al., 2019, Ilioska et al., 2023, Lombardo et al., 2019). A limitation of the infant work to date is that many of the studies were conducted on the same cohort (see starred references in Fig. 1), and thus require replication in new samples. While there is much left to learn about the developing visual system in ASD, findings to date provide compelling evidence that the visual system develops atypically beginning in early infancy and persisting into adulthood.

## 3 Genetic factors and the developing visual system in ASD

### 3.1 Genetic influences on visual system brain development

Like many aspects of brain development, the structure and function of visual circuitry are heritable. Twin studies have reported moderate to high levels of genetic influences on neuroimaging measures of visual system features, including the cross-sectional area of the optic tract (Miyata et al., 2022), surface area of visual cortical areas (Jha et al., 2018, Miyata et al., 2022), sulcal patterning in the occipital lobe (Pizzagalli et al., 2020), and the functional organization of early visual cortical areas (Alvarez et al., 2021, Benson et al., 2022). Further, it has been shown that visual cortical areas (V1–V3) vary substantially (up to ∼ 3-fold) in their size across individuals, independent of total cortical surface area (Benson et al., 2022), which, when considered alongside evidence of high polygenicity (Grasby et al., 2020) and strong genetic influences (Benson et al., 2022, group, E.N.G. through M.-A.C. (ENIGMA)—Genetics working, 2020) in these regions suggests a notable role for inherited factors in controlling the development of the visual system.

Genetic liability for ASD has also been linked to visual cortex development. Widespread transcriptomic changes have been observed across the cortex in ASD, following an anterior-to-posterior gradient with the most robust differences observed in primary visual cortex (Gandal et al., 2022). Several studies comparing the spatial distribution of brain differences in ASD (i.e., functional connectivity, cortical thickness/surface area) to transcriptomic atlases of the human brain have revealed that differences in visual cortex/networks are associated with genes important for regulating excitation and inhibition (Berto et al., 2022) and immune and synapse function (Buch et al., 2023). In line with this, work harnessing the infant sibling study design has linked familial markers of inherited genetic liability for ASD to individual variation in the structure and function of components of the visual system (Girault et al., 2022). Specifically, autistic traits in older ASD siblings (probands) were found to explain up to 20 % of the variation in multimodal aspects of visual circuitry in HL siblings from 6 to 24 months of age, such that higher levels of autistic traits, indicative of greater genetic burden (Frazier et al., 2014, Nijmeijer et al., 2010, Thomas et al., 2021), were positively correlated with HL-ASD sibling surface area in the middle occipital gyrus and the microstructural integrity of the splenium of the corpus callosum. Greater levels of proband ASD traits were also associated with weaker functional connectivity between the visual/medial-visual networks and the DMN at 6 months in HL siblings. Weaker visual-DMN connectivity has been linked to fewer bids for joint attention in an overlapping sample (Eggebrecht et al., 2017) and observed in a separate sample of ASD toddlers with deficits in visual attention to social stimuli (Lombardo et al., 2019). This suggests that genetic liability for ASD, as indexed by quantitative variation in ASD traits among family members, may play an important role in shaping multiple aspects of infant visual system development across the first years of life.

The complex polygenic and pleiotropic genetic architecture of ASD (Cirnigliaro et al., 2023), along with the multimodal changes in the visual system – including cortical surface area expansion, regional brain overgrowth, atypical patterns of myelination and differences in functional connectivity – implicate multiple pathogenic processes. Proposed mechanisms include the expansion of the neural progenitor pool, resulting in increased surface area and brain overgrowth (Marchetto et al., 2017, Packer, 2016, Piven et al., 2017), and experience-dependent processes (i.e., synaptic plasticity) that regulate myelination, cortical expansion, and volumetric growth (Girault and Piven, 2020, Pretzsch and Ecker, 2023). Several ASD genes also converge on molecular signaling pathways, including the Wnt pathway (Hashem et al., 2020), that is responsible for regulating many aspects of embryonic development including cortical development and arealization, especially in the occipital cortex (Grasby et al., 2020). Together, these findings suggest a complex etiology for ASD grounded in genetic influences on early brain development, especially as it relates to the patterning of the cortex, with notable impacts on visual cortex and its early-developing connections to the rest of the brain.

### 3.2 Genetic influences on visual processing and attention

In addition to the role genetics plays in the development of visual circuitry, there is also considerable evidence that aspects of basic visual processing and the allocation of visual attention are also highly heritable. Twin studies and genome-wide association studies (GWAS) have demonstrated that approximately 50 % of the variance in various aspects of visual processing can be accounted for by genetic factors, including binocular rivalry (Miller et al., 2010), visual contour integration (Zhu et al., 2019), and biological motion perception (Wang et al., 2018). In a seminal eye-tracking study in 18- to 24-month olds, Constantino and colleagues reported that twin-twin concordance of visual attention and eye-gaze features was very high among monozygotic twins (0.91) (relative to dizygotic twins, 0.35), meaning that how children viewed social scenes – *down to the millisecond* – was under stringent genetic control (Constantino et al., 2017). The most heritable features of gaze patterns across this age range were found to be preferential attention to eye and mouth regions of the face, which were the same features that were found to be atypical in toddlers with ASD. A study in 5-month-olds downward extended these findings, demonstrating that preferences to eyes is heritable (heritability estimated at 57 %) even earlier in infancy (Viktorsson et al., 2023).

Family studies have also suggested that individual variation in visual attention is influenced by genetic background and genetic liability for ASD, as both non-ASD parents and siblings of children with ASD exhibit atypical visual attention and gaze behavior (Adolphs et al., 2008, Dalton et al., 2007). Even among TD infants and toddlers, subthreshold variations in autistic traits (i.e., social responsiveness and social motivation) in parents is predictive of their attentional profiles. For example, an eye-tracking study reported that typically developing 8-month-old infants born to parents without ASD, but with higher levels of subthreshold autistic traits, exhibit slower visual orienting, which was in turn correlated with later social communication abilities at 21 months of age (Ronconi et al., 2024). Similarly, Jones and colleagues found 6- and 12-month-old infants of parents with lower levels of social motivation exhibited reduced social attention and blunted neural responses to faces during a combined EEG and eye-tracking task (Jones et al., 2016). Taken together, this suggests that population variation in heritable aspects of social motivation may play a driving role in the development of the visual system and its attunement to social information in the environment.

## 4 The infant visual system is a building block for attention, cognition, and social development

The maturation of the visual system is a critical building block to cognition via its fundamental involvement in visual attention, which scaffolds learning during infancy by shaping the environment (Amso and Scerif, 2015, Oakes, 2023, Scarr and McCartney, 1983). There is a growing body of evidence linking individual variation in the structure and function of the visual system to aspects of visual attention and processing, cognition, and social behavior in the first years of life in both ASD and TD.

### 4.1 Infant visual system and low-level visual processing, attention

In a MRI and eye-tracking study in HL infants, Elison and colleagues compared white matter development to performance on the gap overlap eye-tracking task administered in 6–7-month-old HL and TD infants (Elison et al., 2013a). They found that orienting latency (i.e., the time it takes for an infant to disengage and shift attention from one stimulus to another) was delayed in HL-ASD infants compared to TD infants, with HL-Neg infants who did not develop ASD exhibiting an intermediate phenotype. Individual variation in orienting latency was associated with white matter properties (radial and axial diffusivity and fractional anisotropy) in the splenium of the corpus callosum (connecting the bilateral visual/occipital cortex) in TD infants, with HL-ASD infants exhibiting a different brain-behavior coupling. Studies in typically developing infants have linked variations in white matter measured by MRI to low-level visual processing and higher-order attentional behaviors. For example, in a follow-up study in TD infants, Elison and colleagues reported fronto-limbic circuitry involving the amygdala at 6 months was correlated with the number of bids for joint attention at 9 months of age (Elison et al., 2013b). Reports in preterm infants scanned in the neonatal period demonstrate links between basic visual function (i.e., fixation and tracking measured during neurological exam) and the microstructural integrity (Bassi et al., 2008, Berman et al., 2009) or development (Groppo et al., 2014) of the optic radiation. Similarly, a study using electroencephalography (EEG) to measure visual evoked potentials (VEP; an event-related response to visual input that follows a known developmental pattern (Crognale et al., 1997; McCulloch et al., 1999)) found strong correlations between VEP metrics and MRI-derived white matter properties specific to the optic radiation (Dubois et al., 2008). Together, these findings suggest an important role for developing white matter in subserving variation in basic visual processing and higher-order attention behaviors during the first months of life.

A MRI functional connectivity study in 12–48-month-olds with ASD reported weaker connectivity between the DMN and occipital-temporal cortical (OTC) areas in a subset (∼ 30 %) of the ASD sample (Pierce et al., 2015) with pronounced visual social engagement difficulties as determined by eye-tracking (Lombardo et al., 2019). Among this ASD subset, individual variation in OTC-DMN connectivity was associated with social communication difficulties measured by standard clinical assessments, suggesting that weakened OCT-DMN functional connectivity in toddlerhood may give rise to observable clinical features via visual attentional processes. This aligns with evidence in a combined sample of HL and TD infants that weaker connectivity between the visual network and the DMN was correlated with fewer bids for joint attention at 12 months of age during a play-based laboratory assessment (Eggebrecht et al., 2017).

While these studies highlight the ability to detect links between early brain measures and visual processing and attention, there remains a critical need to identify and understand developmental (i.e. longitudinal) circuit-based neural substrates subserving visual behaviors across the infancy period and their implications for downstream brain and behavior development. This is especially relevant given hypotheses regarding developmental shifts in circuitry supporting oculomotor and attention behaviors in the first months of life (***see*** Section 6.1).

### 4.2 Infant visual system and cognition

Several studies have linked visual system development during infancy to variations in downstream cognitive abilities. For example, a MRI study of developing neural flexibility – the frequency at which brain regions change their membership from one functional module to another – found that the primary visual network was uniquely stable (compared to motor and higher-order networks) across the first two years of life and that reduced flexibility at 3 months was related to higher overall cognitive ability at 5–6 years of age (Yin et al., 2020). Behavioral studies also suggest an important role for the infant visual system in cognitive development. Visual tracking (measured by electrooculography) at 4 months of age in infants born very preterm have been reported to predict of overall cognitive abilities measured at 3 (Kaul et al., 2016) and 6.5 years of age (Kaul et al., 2022), independent of visual function. Similarly, a study of both preterm and term-born infants found that visual fixation and tracking abilities (measured during neurological exam) in newborns was correlated with visuo-motor abilities (i.e., hand-eye coordination, pattern recognition) at age 2 and non-verbal cognition at age 5 (Stjerna et al., 2015). Together, this suggests that visual system development in the first months of life may influence trajectories of learning and cognition abilities which rely on rapid processing of visual input.

### 4.3 Infant visual system and social behavior

Prior work in HL and HL-ASD infants has demonstrated links between atypical patterns of visual system brain development and impairment in social behavior during the first years of life. Shen and colleagues reported that faster rates of amygdala volume growth on MRI from 6 to 12 months of age in HL-ASD infants were correlated (r = 0.52) with greater social deficits measured at 24 months (Shen et al., 2022); findings were specific to social behavior, as the authors reported no correlation with repetitive behaviors. Nair and colleagues reported that, in a sample of HL infants, thalamic-occipital functional connectivity strength measured by MRI at 6 weeks of age was correlated with autistic traits (r ≥ 0.55) and symptoms endorsed by both parents and clinicians at 36 months of age (Nair et al., 2021). Similarly, as noted above, Lombardo and colleagues reported strong associations between atypical DMN-OTC MRI functional connectivity and social deficits (r = -0.78) in a subset of ASD toddlers with reduced visual attention to social stimuli (Lombardo et al., 2019). The strength of correlations between visual brain circuitry and social behavior are notable, as minimal (r = 0.17) (Hazlett et al., 2017) to no correlation (Amaral et al., 2017, Hazlett et al., 2005) has been observed between social behavior and global brain overgrowth in ASD during toddlerhood and preschool. These findings in infancy and toddlerhood align with MRI studies in adults and children linking the structure and function of the visual system to social behavior and autistic traits (Buch et al., 2023, Fishman et al., 2018, Mazziotti et al., 2022).

## 5 A developmental cascade model involving the visual system in ASD

The developmental cascades framework proposes that development is a cumulative consequence of interactions across domains, biological systems, and environmental contexts that fundamentally alter the course of development (Masten and Cicchetti, 2010). It has been applied to neurodevelopmental disorders including ASD (Bradshaw et al., 2022a) and genetic syndromes (Järvinen-Pasley et al., 2008) and provides an important framework for describing how initiating events may spread across multiple systems of development (i.e., from one-to-many neural circuits, from a lower-level to many higher-order behaviors).

Following this framework and building from the evidence regarding atypical visual system development in ASD, we hypothesize that genetic liability for ASD initiates the atypical ontogenetic development of the visual cortex and its connections with other brain areas (e.g., thalamus, amygdala, extrastriate areas), resulting in differences in the macroscale structure and function of visual circuits detectable in the first weeks and months of life in HL and HL-ASD infants. These differences in visual circuitry give rise to deficits in low-level visual processing and attention that emerge in the months after birth. The impact of atypical attention behaviors is likely to be two-fold, influencing the development of downstream cognition and social behavior by both (1) constructing ecological niches for learning (Constantino et al., 2017, Scarr and McCartney, 1983) and (2) leading to the continued atypical development of the visual system and its connections with other brain networks though experience-dependent processes for circuit refinement during infancy, fundamentally altering cortical hierarchies and functional network topologies (Hong et al., 2019) for higher-order cognitive processing, and potentially leading to inefficient circuit refinement and the overgrowth of the brain (Piven et al., 2017). This cascade of brain-behavior changes may ultimately give rise to aspects of the ASD phenotype, and in particular its defining social features, later in toddlerhood by disrupting both the neural circuitry and environmental context for social learning.

While the initial evidence for this model is compelling, there are many critical components of the cascade that have yet to be tested, and no work to date has considered the entire model. Determining how visual circuitry develops to support visual processing, attention, and social behavior during a period of heightened plasticity and vulnerability during the infancy period is a defining research agenda for future work.

## 6 Critical gaps in our understanding of human visual system development

Our understanding of the visual system is largely derived from studies in awake adults, non-human primates, and animal models. Far less is understood about visual system maturation in human infants, especially during the first months of postnatal development. While there have been several pioneering neuroimaging studies of visual system structure (Dubois et al., 2008, Groppo et al., 2014, Natu et al., 2021, Stjerna et al., 2015) and function (Biagi et al., 2023, Biagi et al., 2015, Deen et al., 2017, Ellis et al., 2021) in early infancy, most include largely cross sectional or relatively sparse longitudinal sampling, and none have jointly examined subcortical and cortical components of visual circuitry or links to later behavior. Findings to date in HL and HL-ASD infants suggest that some aspects of visual circuitry exhibit atypical development in the first 6 weeks (Nair et al., 2021) to 6 months of life (Elison et al., 2013a), aligning with evidence of differences in visual attention that emerge in the early postnatal period (Bradshaw et al., 2019, Giorgio et al., 2021, Giorgio et al., 2016, Jones and Klin, 2013). Together, this calls for a detailed understanding of how the human visual system develops to support visual attention in the first months of life. We focus specifically on two key areas for future research: (1) defining the neural circuitry that underlies visual attention development in the first six months of life and understanding links to later, higher-order circuit maturation, and (2) understanding the role of visual processing in the modulation of attention during infancy.

### 6.1 Shifts from subcortical to cortical modulation of visual behavior in infants – the critical need to examine this hypothesis

While the exact timing of the onset varies somewhat between studies, there has been consistent evidence that visual attention is atypical across the first months of life in ASD and HL infants (Bradshaw et al., 2022b, Bradshaw et al., 2019, Giorgio et al., 2021, Giorgio et al., 2016, Jones and Klin, 2013), with differences that are prominent and detectable across multiple cohorts by approximately 6 months of age (Chawarska et al., 2013, Elison et al., 2013a, Jones et al., 2016, Jones and Klin, 2013). It has been hypothesized that differences in visual attention in ASD during the first months of life are related to a “developmental shift” from subcortically- to cortically- mediated visual behavior (Johnson, 1990, Johnson, 2005, Johnson et al., 2015a, Johnson et al., 2015b), such that earlier-acting subcortical circuits involving the amygdala, pulvinar, and superior colliculus are initially “intact”, but that the shift to cortically-mediated routes (lateral geniculate nuclei to visual cortex, visual cortex to dorsal/ventral pathways) later in infancy is delayed or disrupted (Johnson, 2005, Johnson et al., 2015a, Johnson et al., 2015b, Jones and Klin, 2013, Klin et al., 2015). However, to date, no brain-behavior studies provide empirical evidence to directly support this hypothesis in ASD, nor are there any published studies in human infants documenting this shift occurs in TD. In fact, there is an active debate in the field as to the relative contributions of subcortical and cortical pathways to gaze behavior and other functions (e.g., motor control, cognition) in infancy (Aslin et al., 2023, Blumberg and Adolph, 2023, Liu et al., 2023), driven by mounting evidence that the visual cortex and cortical areas involved in visual processing are functional during visual processing in early infancy (Biagi et al., 2023, Biagi et al., 2015, Deen et al., 2017, Ellis et al., 2021). This has resulted in multiple calls to investigate this hypothesis developmentally (Constantino, 2018a, Klin et al., 2015, Liu et al., 2023).

A developmental approach is further bolstered by evidence from animal models suggesting that there may be transient connections between subcortical (e.g., pulvinar) and cortical visual areas in early development that could play a fundamental role in not only the early processing of visual input, but also the patterning and development of the visual cortex (Bridge et al., 2016). Studies that involve state of the art neuroimaging at dense sampling rates during infancy have the incredible potential to reveal patterns of structural and functional connectivity between subcortical and cortical circuits across this period to inform the current understanding of postnatal visual system development. Further, empirical studies linking subcortical and cortical activity to looking behavior in infants younger than 6 months of age will be necessary to test the relative contributions of each pathway to gaze behavior and visual attention (Liu et al., 2023).

It will also be essential to understand how the early-developing visual system may scaffold the development of higher-order networks that support cognition and learning (Rosen et al., 2019). For example, brain overgrowth is observed after a period of atypical early visual system development in ASD (Hazlett et al., 2017), and atypical cortical hierarchies involving the visual system are observed in ASD (Hong et al., 2019), yet it remains unclear whether (or to what degree) differences in the development of the visual system are causal or specific to these downstream differences in global aspects of circuit development or if they are the bellwether of what is to come as the rest of the brain matures. While it has been hypothesized that the prefrontal cortex, for example, develops independently of sensory input (Johnson et al., 2015a, Johnson et al., 2015b), these ideas have been more recently challenged by work in animal models demonstrating that activity in the developing prefrontal cortex is modulated by sensory experience (Gómez et al., 2023). In humans we may be able to test the role of visual input on downstream circuit changes through experimental manipulations designed to stimulate the developing visual system, with some examples described below (Section 7).

### 6.2 The role of visual processing in modulating attention

Differences in visual attention may be subserved or accompanied by deficits in low-level visual processing. Indeed, differences in various aspects of low-level visual processing have been captured by EEG during infancy, childhood, and adulthood in ASD (Hardiansyah et al., 2023, Richards et al., 2023, Siper et al., 2016) and related neurodevelopmental disorders (Gallego et al., 2014, Lalancette et al., 2022). This includes binocular rivalry, which occurs when visual perception alternates between incompatible images presented to each eye and is reported to be slowed or fundamentally altered in ASD by adulthood (Spiegel et al., 2019). Differences in motion perception measured by EEG have also been well documented (Spiteri and Crewther, 2021), with recent evidence of atypical motion processing as early as 5 months in HL infants (Hardiansyah et al., 2023, Nyström et al., 2021). Luminance contrast sensitivity measured by EEG also differs in 6-month-old HL infants compared to TD infants (McCleery et al., 2007), which may arise via atypical function of the magnocellular (M) pathway that carries visual information to the superior colliculus and pulvinar as part of a “quick and dirty” processing stream for high-level features consistent with general elements human face configurations (i.e., eyes, nose) (Johnson, 2005). M pathway function measured using MRI has also been reported to be atypical in adults with ASD (Schelinski et al., 2024), while EEG has captured differences in males with fragile X syndrome, an X-linked single-gene disorder that shares many clinical features with ASD (Kogan et al., 2004). Interestingly, several aspects of visual processing captured by eye-tracking have also been shown to differ between males and females (Shaqiri et al., 2018), as well as the development of contrast sensitivity across infancy measured by EEG (Dobkins et al., 2009). This aligns with MRI evidence that visual cortical areas responsible for object recognition and face processing are among the most sexually dimorphic structures in the brain (at least in terms of size) (Liu et al., 2020) and suggests an interesting sex effect on the developing visual system with relevance to ASD that deserves further study.

Many visual processing abilities have been shown to develop in an experience dependent manner in EEG studies, such that increased postnatal visual experience (quantified by comparing infants born at and before term matched for gestational age) impacts the rate of development (Dobkins et al., 2009, Jandó et al., 2012). This highlights the powerful role of early experience in shaping these low-level processing abilities, highlighting their potential as modifiable targets for very early intervention. While some preliminary evidence (Amso et al., 2014) exists to suggest that visual skill development in infants may guide the development of visual attention, very little work has been done to comprehensively map the development of visual function and downstream visual attention *within the same individuals* over time. This will be essential to understanding how the earliest stages of visual development may serve as a physiological scaffold, or filter, on what infants can resolve and attend to in the visual field (Purpura and Tinelli, 2020).

## 7 The visual system: measuring developmental change, promise for intervention

Classical studies of experience-dependent plasticity show profound effects on local circuitry in the visual cortex after periods of visual deprivation during critical periods of development, as well the remarkable ability to modulate or “re-open” the critical period to rescue certain visual functions and visual processing deficits (Hensch, 2005). This both highlights the potential vulnerability and adaptability of the visual system as a target for very early intervention in HL infants, and suggests that the visual system may be easily modulated to examine the developmental cascade model. There are interesting and compelling examples with relevance to ASD, including interventions targeting visual processing shortly after birth as well as training of visual attention later in infancy. The intervention and training studies described below highlight the potential to examine developmental cascades in the visual system by modulating visual and visual-attentional experiences.

Several studies in infants find that interventions in the first weeks of life using multisensory stimulation induce changes in basic visual processing measured by EEG (Blystad and Meer, 2022, Fontana et al., 2020, Guzzetta et al., 2009, Rodovanski et al., 2021). For example, Guzzetta and colleagues tested whether infant massage in the neonatal intensive care unit accelerated brain development in infants born preterm (30–33 weeks), and then performed a cross-species comparison in rats to examine underlying mechanisms of action. They found that preterm infants (n = 10) who underwent postnatal massage (15-minute sessions performed 3 times a day for 10 days over a 12-day period) displayed evidence of more mature VEP signals (N300 latency) and increased visual acuity at 3 months of age compared to control infants. Similarly, massaged rat pups also showed more mature VEP responses compared to controls, which was mediated by levels of IGF-1, a growth factor that regulates brain development (when IGF-1 was blocked, there were no effects of massage). The authors conclude that tactile stimulation produces changes in brain development, modulated by IGF-1, most notably (but not only) in visual cortex which is developing rapidly during a critical period. Similarly, Blystad and colleagues recorded VEPs at two timepoints in infancy, first around 4–5 months of age and again after experience with self-locomotion (e.g., crawling) between 9 and 12 months of age in infants who received extra motor stimulation in the form of weekly infant swim lessons (beginning at 1.5–4 months of age) with a caregiver compared to two other groups without extra stimulation (a full-term and preterm group). They found that at both the first and second visits, infants who received extra motor stimulation exhibited enhanced development of visual motion perception, and also had the greatest developmental improvements in visual motion perception over time, indicated by VEP N2 latency (Blystad and Meer, 2022). These findings fit with a large body of evidence that action-perception associations are essential to early brain development (Byrge et al., 2014, Jayaraman et al., 2015), and suggest that relatively simple interventions that may be feasibly implemented in the home (Rodovanski et al., 2021) have the potential to induce changes in low-level visual processing. These studies also provide preliminary evidence of readouts of intervention success in the form of EEG recordings; future efforts should consider using MRI to understand the underlying circuit changes, including changes in white matter which happen rapidly across this period and signal underlying changes in plasticity (Swanson and Hazlett, 2019).

A series of studies have suggested that training visual attention using eye-tracking paradigms in older infants (9–12 months) is feasible and can impact social responsiveness. In five laboratory visits over 15 days, Wass and colleagues demonstrated that attentional training (using eye-tracking, screened based protocols) in 11-month-olds led to improved cognitive control and sustained attention, as well as reduced saccadic reaction times (Wass et al., 2011). Implementing a similar training paradigm in community settings meant to engage lower-resource families resulted in largely similar findings to their original lab-based study, suggesting feasibility outside of strictly controlled settings, though drop-out rates were quite high (Ballieux et al., 2016). Finally, a six-week attentional training period was tested for its transfer effects to more complex social and cognitive skills during infancy, with training starting at 9 months of age (Forssman and Wass, 2018). The authors reported significant increases in sustained attention both immediately following training and at 6-week follow-up, as well as increases in responsiveness to social cues (following pointing and gaze) at follow-up, with training gains correlated with the degree of improvement in social responsiveness. Together, these findings suggest that training visual attention during the later part of the first year of life can result in changes in attention and social responsiveness, though the long-term effects have yet to be examined/reported. Future work is needed to understand whether these interventions may be implemented outside of controlled experiments with specialized equipment, or what changes they may produce, if any, in HL infants who already show differences in visual attention by these ages.

While the field is far from implementing any specific interventions for visual system development in HL infants, each of the examples above provides important preliminary evidence that the visual system is adaptable, and that changes in visual processing and attention can be measured after brief periods of training or intervention during infancy and toddlerhood. Future work in infants should consider how such paradigms may be incorporated into longitudinal developmental research to directly examine cascades linking the visual system to downstream brain and behavior development.

## 8 Conclusions and the path forward

**Fig. 2:**
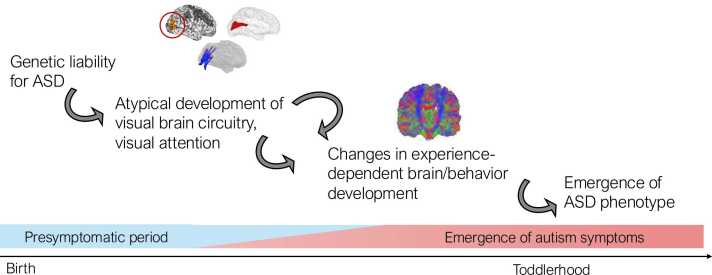
Developmental cascade involving genetics, visual system circuitry, visual attention, and downstream brain development, cognition and behavior in ASD. In this hypothesized cascade model, genetic liability for ASD influences the development of visual circuitry that subserves atypical visual attentional and gaze behavior during infancy. Atypical visual system function and differences in visual attention create an altered visual environment for learning, impacting experience-dependent neural development and the development of higher-order brain networks that subserve cognition and social behavior, ultimately giving rise to widespread differences in brain development (e.g., brain overgrowth) and the ASD phenotype that emerges during toddlerhood.

The development of the visual system may have a fundamental role in the emergence of ASD that warrants further study, though several outstanding questions must be addressed (Box 1). These include the need for a more detailed understanding of the neural circuitry supporting visual attention across the first months of life and a mapping of the associations between low-level visual processing and attention across infancy. Prospective studies will be essential to addressing the gaps in knowledge and will need to include multiple levels of analysis (e.g., MRI, EEG, eye-tracking, naturalistic behavior, clinical assessment) and dense longitudinal sampling (e.g., monthly, or on a scale similar to expected intervention timelines). Future work should also consider testing the developmental cascade model directly (Fig. 2) through experimental trials stimulating visual system development and measuring downstream changes in both brain and behavior across proximal (weeks) and distal (several months) time points, including longer-term follow-up to assess impact on adaptive behaviors (e.g., social ability, communication skills). These initial studies need not take place solely in HL infants; important evidence of the developmental cascade could be confirmed in typically developing samples first, setting the stage for rigorous studies to follow in HL samples.

Box 1 **Questions for Future Study**

• What are the relative contributions of subcortical and cortical visual circuitry to visual processing and attention in human infants?
• How does low-level visual processing during the first months of life map to changes in attention and how are these linked to brain development in typically developing and HL infants?
• Are there sex differences in the development of the visual system during infancy that may underlie the higher occurrence of ASD in males?
• What role does genetic background and genetic liability in play in shaping variation in the developing visual system in both typical development and ASD?
• Can interventions targeting low-level visual processing promote adaptive behavioral development?

The next wave of longitudinal studies aimed at examining visual system development in both TD and HL samples should make every effort to examine the role that background genetics and biological sex may play in the emergence of phenotypic variation in visual system neurobiology, visual processing, attention, and later social behavior – without a careful consideration of these factors in both cases and controls, we may miss important details about causal influences in ASD (Constantino, 2018b). Background genetics and genetic liability can be indexed behaviorally in family members, with detectable effects in relatively small samples, making this a feasible addition to many study protocols (Girault et al., 2022, Girault et al., 2020, Jones et al., 2016, Ronconi et al., 2024). Finally, the consideration of a developmental cascades framework involving the visual system as a building block and intervention target has the potential to inform not only etiology and intervention targets idiopathic ASD, but also for genetically related neurodevelopmental disorders (e.g., ADHD) with known atypicalities in visual system neurobiology, attention, and processing (Vandewouw et al., 2020).

## Funding Information

Dr. Girault’s work is supported by the 10.13039/100000002National Institutes of Health (K01-MH122779; R01-HD055741; R01-MH123716, R01-MH130441), the 10.13039/100008152Autism Science Foundation, and the Foundation of Hope.

## CRediT authorship contribution statement

**Jessica B Girault:** Writing – review & editing, Writing – original draft, Conceptualization.

## Declaration of Competing Interest

The authors declare that they have no known competing financial interests or personal relationships that could have appeared to influence the work reported in this paper.

## Data Availability

No data was used for the research described in the article.
